# Spatially Resolved Ion Sensing by Voltammetric Ion Transfer Microscopy

**DOI:** 10.1021/jacsau.5c01034

**Published:** 2025-10-21

**Authors:** Gabriel J. Mattos, Justine A. Rothen, Thomas J. Cherubini, Eric Bakker

**Affiliations:** Department of Inorganic and Analytical Chemistry, 34326 University of Geneva, Quai Ernest-Ansermet 30, CH-1211, Geneva, Switzerland

**Keywords:** Electrochemical imaging, TEMPO, Quantitative mapping, Fluorescence, Ion-selective membrane

## Abstract

The visualization and mapping of ionic species in solution and near surfaces are important to understand chemical gradients and spatially resolved dynamic processes in various fields. Available label-free approaches are either slow or restricted to a few parameters, such as pH. We introduce here a novel chemical mapping principle for the spatially resolved sensing of optically silent ionic species at high frequency, acquiring a concentration map of millions of pixels in seconds using a conventional fluorescence microscope. The principle relies on ion transfer from a thin polymeric film into a solution phase, electrochemically coupled to electron transfer at the back side of the film. Different solution concentrations change the potential at which ion transfer is observed, which is visualized by unquenching a fluorophore when the redox probe in the film is electrochemically oxidized. The moment of maximum fluorescence change for each pixel is captured by a rapid image burst to simultaneously find the excitation peak potentials for all pixels. This produces a concentration map, turning a single sensing film into a chemical imaging platform that provides millions of concentration points. The imaging principle is demonstrated with a flowing junction to map diffusional mixing of two solution streams with different ion concentrations, using tetraethylammonium as an initial model ion, to achieve micrometer spatial resolution.

## Graphical abstract

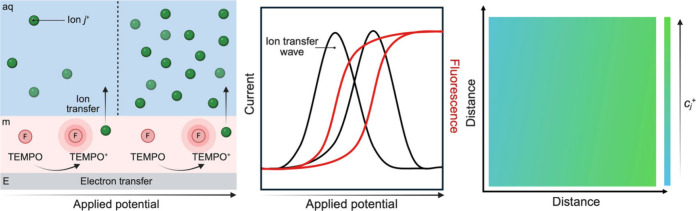

## Introduction

Scanning probe microscopy was first introduced as scanning tunneling microscopy (STM) and atomic force microscopy (AFM) by Binnig and co-workers to achieve topographical imaging at the atomic scale.^1,2^ With scanning electrochemical microscopy (SECM), Bard subsequently combined this approach with electrochemistry to obtain spatially resolved chemical information at the microscale.^3^ SECM relies on an ultramicroelectrode that scans across the surface while measuring faradaic currents or potential.^4–6^ In an alternative approach, light-addressable potentiometric (LAPS) and amperometric sensing use a scanning light beam to generate localized photocurrents that may change in surface potential.^7–9^ A recent example is scanning electrochemical cell microscopy (SECCM), which uses nanopipettes as scanning probes at surfaces.^10,11^ All of these techniques allow for the detailed imaging of electrochemical activity and spatially resolved reactions near surfaces. Owing to the need to move a probe across the surface, such techniques require specialized instrumentation and are often too slow for the mapping of larger (area > mm^2^) and highly dynamic systems.^12,13^ For example, imaging a single 3 million-pixel image at a typical scan rate of 1 Hz (1 pixel s^–1^) would require as many seconds or about 1 month, which is impractical. In contrast, we introduce here a complementary method that can achieve higher throughput, generating such an image in just a few seconds.

Optical sensors may also provide spatially resolved optical detection.^14,15^ Such optodes are typically polymeric films that respond to target solution analytes by producing changes in color or fluorescence, allowing concentration heterogeneities to be visualized with optical imaging tools including consumer cameras.^16–18^ Adequate optical sensors for this application have mainly been developed for O_2_ and pH mapping.^19–21^ Sensors for other ionic species have been reported,^22,23^ but the underlying cross-response to pH of these optodes does not currently make them sufficiently attractive as chemical imaging tools.^24^

Recent advances have also demonstrated operando optical imaging of electrochemical systems to visualize ion transport and redox activity.^25,26^ While powerful, these studies rely on optical signatures that arise either from refractive index changes or from the absorbance of optically active redox species. We introduce here a novel opto-electrochemical technique (see Figure 1a) that allows one to image optically silent, non-redox-active ionic species in solution without a label.

**Figure 1:**
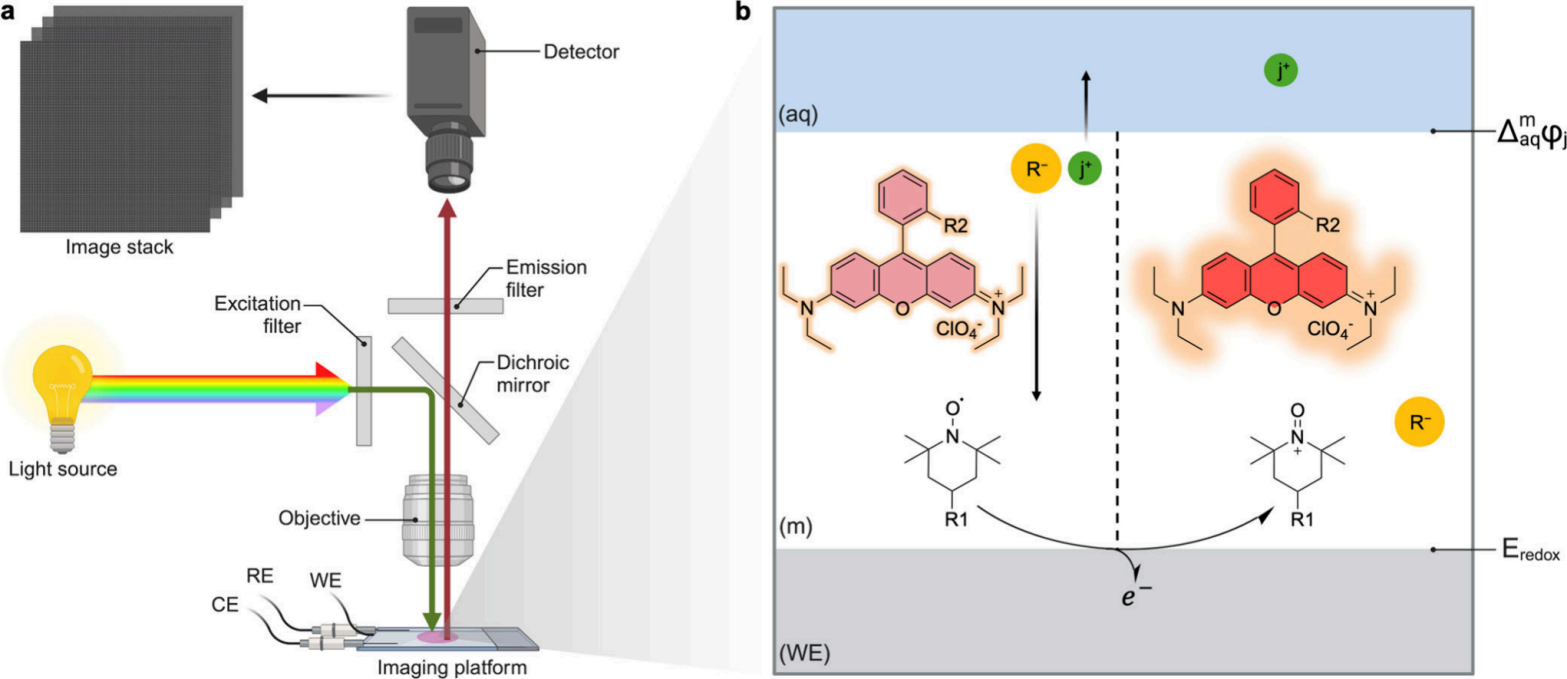
Imaging setup and working mechanism. (a) Schematic diagram of the opto-electrochemical system. The imaging platform is an electrochemical glass slide cell consisting of working (WE), reference (RE), and counter (CE) electrodes. The cell is placed under a wide-field fluorescence microscope and connected to a potentiostat. Excitation and emission filters are set for rhodamine imaging, and the high-resolution camera generates an image stack of the membrane surface at a certain frequency as the potential is scanned at a defined rate. (b) Imaging principle. The sensing phase on top of the WE consists of a thin polymeric membrane (m) containing an ion exchanger (R^–^), lipophilic TEMPO (R1 represents a heptadecyl ester) in its neutral radical form, and a lipophilic derivative of rhodamine (R2 represents an octadecyl ester). The electrochemical conversion of the radical form (TEMPO*) into the corresponding oxoammonium cation (TEMPO^+^) results in transferring a cationic species *j^+^* from the membrane to the aqueous phase. Simultaneously, it triggers the fluorescence intensity of rhodamine, since the cationic form of the TEMPO redox mediator quenches the dye to a lower extent.

The new principle is shown in Figure 1 and uses a single-polymer-modified electrode to obtain millions of concentration pixels in a matter of seconds. The method relies on the optical visualization of the electrochemical turnover of a redox probe embedded in a thin polymer film that overcoats the electrode. The oxidation of this probe is dictated by the applied potential but additionally modulated by the ion concentration (strictly, activity) in solution. This modulation is the key feature that allows for the technique to become an ion imaging principle. The oxidation of the probe must be coupled to the expulsion of a cationic species from the film to maintain charge balance (Figure 1b). A higher concentration of the cation in solution makes it energetically more difficult to be expelled from the film. Consequently, for a given applied potential, the potential available for oxidation becomes smaller with a higher ion concentration in solution.^27^ For a heterogeneous ion distribution in an aqueous sample, a potential sweep at the electrode results in a range of oxidation peak potentials for the redox probe. An optical imaging readout is now required to visualize all individual redox probe transitions at pixel-level resolution. This is accomplished with a co-localized dye in the film that becomes unquenched upon oxidation of the redox probe. The potential scan is accompanied by a rapid image acquisition burst that allows one to identify the potential of the largest fluorescence change for each pixel. From this information, an ion concentration map in solution can be obtained.

Recently introduced by our group, lipophilized tetramethylpiperidine *N*-oxyl (TEMPO), a stable nitroxide radical containing a lipophilic heptadecyl ester chain to retain it in the membrane, serves as a redox mediator for ion sensing in ion transfer voltammetry.^28,29^ Its neutral radical form may quench the fluorescence of an appropriate dye such as rhodamine.^30–32^ Electrochemical switching of the redox state of TEMPO to its oxidized form results in an unquenching of the optical reporter dye, allowing one to identify the redox potential by fluorescence imaging. We coin this approach voltammetric ion transfer microscopy (VITM), demonstrate its operational principle in a model system, and compare the experimental results with theoretical predictions.

## Results and Discussion

### Chemical Ion Imaging Mechanism

A lipophilic derivative of rhodamine was selected as the optical probe (for the structure, see Figure 1b) to prevent leakage from the sensing film during measurement. The optical properties of the probe remain similar to those of unmodified rhodamine (absorption maximum at 550 nm and emission at 570 nm, Figure 2), compatible with standard fluorescence microscope filters.

**Figure 2:**
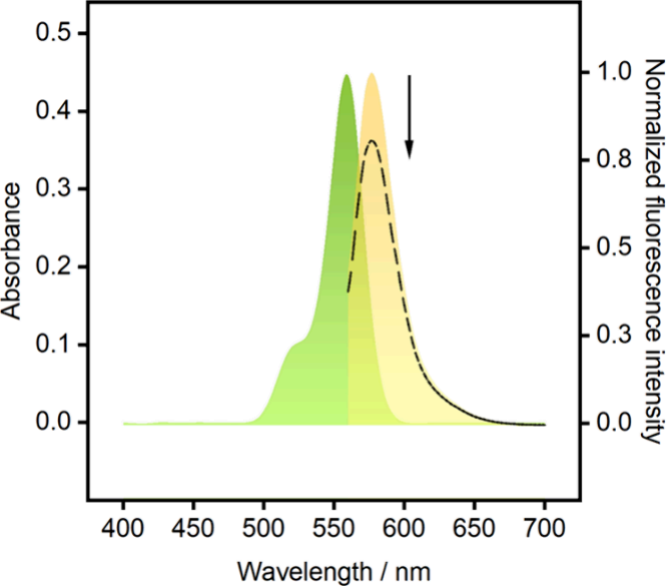
TEMPO quenching effect on rhodamine. Absorbance (green curve) and emission spectra (yellow curve) for the lipophilic rhodamine (5 μmol L^–1^, in tetrahydrofuran) in the absence and presence (dashed line) of the TEMPO redox probe (10-fold excess).

In the presence of the TEMPO radical in THF, about 20% of rhodamine fluorescence becomes quenched (see dashed line in Figure 2). The quenching process is highly distance dependent and should be more efficient in surface-confined systems like the one described here, where both optical reporter and quencher are homogeneously distributed within a polymeric membrane only a few hundreds of nanometers thick. Indeed, when in its radical form in the polymeric sensing phase, TEMPO quenches rhodamine fluorescence by 50% compared to the control without TEMPO (Figure S1).

To enable electrochemical control of the sensing phase, the membrane cocktail consisting of a PVC-based ion-selective matrix, lipophilic TEMPO, a cation exchanger (R^–^), and rhodamine is spin-coated onto an optically transparent conductive indium tin oxide (ITO) modified glass substrate. Upon applying an anodic potential sweep, TEMPO undergoes a reversible one-electron oxidation to form the oxoammonium cation^28,33^ as illustrated in Figure 1b. The oxidized TEMPO acts as a counterion to the negatively charged cation exchanger, prompting the transfer of the cation *j* ^*+*^ from the film into the aqueous phase to maintain electroneutrality (Figure 1b). This process is characteristic of ion-transfer voltammetry, where the voltammetric peak position is governed by both the redox potential of TEMPO (*E*_*redox*_) and the phase-boundary potentials (Δ_*aq*_^*m*^ **φ**),^34^ as depicted in Figure 1b.

Tetraethylammonium (TEA^+^) was chosen as model cation *j* ^*+*^ in this work, but the principle proposed here broadly applies to other ions of physiological and bioanalytical relevance.^35–39^ Figure 3a presents ion-transfer voltammograms for five different TEA^+^ concentrations in the aqueous sample phase. Since a Ag/AgCl wire was used as a reference element, the chloride concentration was kept constant at 25 mM MgCl_2_ while changing the concentrations of TEA^+^ as nitrate salt. At lower concentrations (e.g., 0.1 mmol L^–1^ TEA^+^, blue trace), the transfer of TEA^+^ from the organic film into the solution requires less energy, resulting in a voltammetric peak at lower potentials. As the TEA^+^ concentration increases, the energy barrier for ion transfer increases, shifting the peak potentials to more positive values. The Nernst equation gives that each 10-fold increase in concentration (strictly, activity) should result in a Nernstian shift of 59.2 mV, which agrees well with the value of 59.8 ± 0.8 mV found experimentally (see Figure S2).

**Figure 3:**
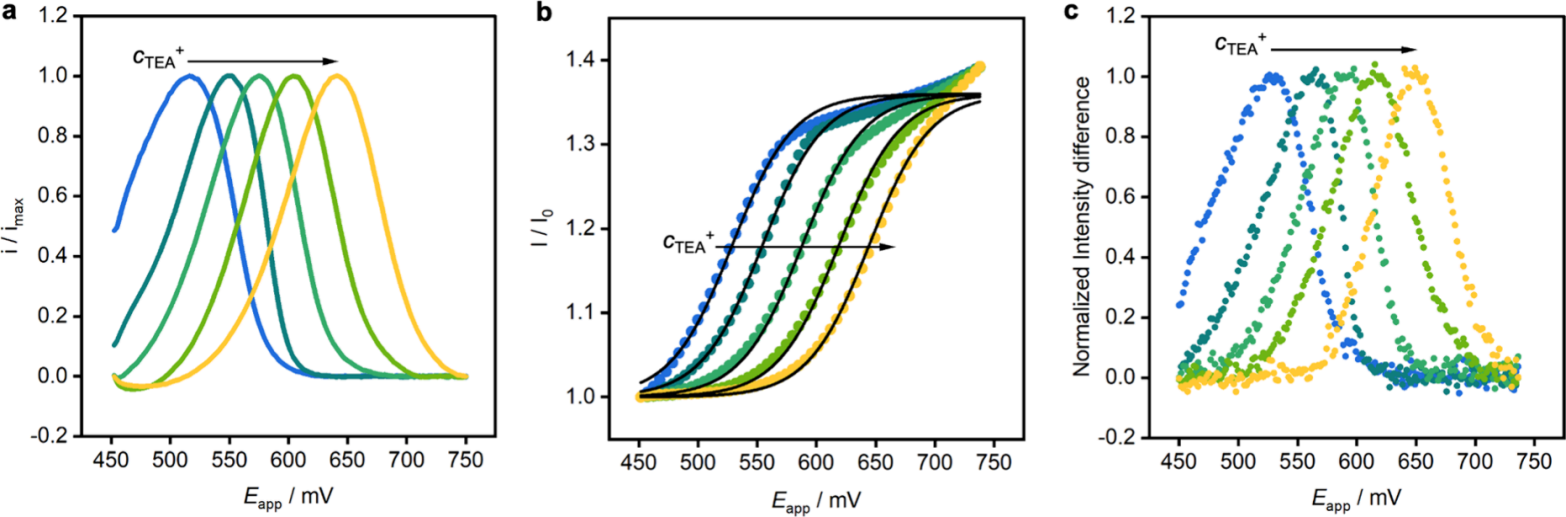
Opto-electrochemical curves for the model ion tetraethylammonium. (a) Linear sweep voltammograms from 450 to 750 mV (vs Ag/AgCl) for changing concentrations of tetraethylammonium in solution, ranging from 0.1 mmol L^–1^ (blue curve) to 10 mmol L^–1^ (yellow curve) with half-order of magnitude increments. The scan rate is 15 mV s^–1^ and the background electrolyte is a 25 mmol L^–1^ MgCl_2_ solution. (b) Colored dots are the average fluorescence intensity of each image of the ion-selective membrane as a function of the applied potential for the corresponding concentrations of tetraethylammonium. Solid lines are the fitting curves based on the idealized model for each ion concentration. (c) Change in the normalized average fluorescence intensity (all pixels) between consecutive frames during the potential sweep for the five different ion concentrations in the sample solution.

The surface confinement of TEMPO ensures that mass transport is not rate-limiting, facilitating its complete electrochemical conversion, a condition achievable only in thin-film ion-selective membranes.^40^ Such thin polymeric films deposited on solid substrates are understood to behave as a liquid phase homogeneously distributed across the surface, as has been previously studied and characterized.^41–44^ As shown in Figure 3a, the current is not diffusion-limited and the half-peak width of 105 mV indicates exhaustive redox conversion.^34^ To regenerate the TEMPO radical form, a reducing potential step is applied between successive anodic scans to allow for multiple cycles with excellent reproducibility as reported earlier.^28^

A rapid image burst of the sensing film during the potential sweep is captured using a high-resolution camera while exciting the optical probe at 550 nm. Figure 3b shows the normalized average fluorescence intensities for the image stack, with the associated current responses in Figure 3a. As can be seen, the average fluorescence intensity of each frame changes with applied potential, reflecting the electrochemical conversion of TEMPO and its quenching effect on rhodamine. Since the optical transition follows the electrochemical conversion coupled to the ion transfer process, these curves shift with the TEA^+^ concentration in solution.

The ratio between the changing fluorescence and the initial intensity (*I*/*I*_0_) follows the reciprocal form of the Stern–Volmer relationship,^45^ which describes intermolecular fluorescence deactivation in the presence of a quencher. This equation resembles a sigmoidal function, which can here be described by combining the electrochemical conversion of the quencher (TEMPO radical) as a function of overpotential (*E*_*app*_ *– E* ^0^) according to the Nernst equation with fluorescence intensity as follows:

1 $$\frac{I}{I_{0}}=A+\frac{B}{1+\frac{1}{c_{j^{+}}}e^{E_{app}-E^{0}/s}}$$

where *A* is the initial value for the ratio (*I*/*I*_0_), *B* is the total change of the fluorescence intensity ratio, *c*_*j*_^+^ is the ion concentration in solution, *E*° is the formal potential, and *s* is the Nernstian slope of 59.2 mV (see Supporting Information for details). Fitting curves from eq 1 are shown in Figure 3b (solid black lines) for the different TEA^+^ concentrations with good correspondence to the experimental data.

The entire image analysis workflow was automated using a mathematical script that analyzes the image sequence in the stack (Supporting Information). The change in fluorescence intensity between consecutive frames (*n* and (*n* – 1)) in the image stack is determined through sequential subtraction according to eq 2,

2 $$I_{diff}=I_{n}-I_{n-1}$$

which enables the identification of the frame corresponding to the maximum intensity difference (*I*_*diff*_). Figure 3c shows the average fluorescence intensity difference (across all image pixels) between consecutive frames during the potential sweep. The largest intensity difference agrees with the voltammetric peak potential, reflecting the electrochemical consumption of the quencher at a specific potential, which depends on the ion concentration in solution. In this manner, the observed optical transition can be quantitatively linked to the local ion concentration via the Nernst equation. Indeed, the optical calibration curve with the peak potentials shown in Figure 3c gives a Nernstian slope of 59.1 ± 0.4 mV and agrees with the electrochemical calibration line shown in Figure S2.

To allow for ion concentration mapping, a difference analysis of consecutive frames is applied to every pixel. Figure 4a shows an image stack used to demonstrate the generation of a chemical map, where each image has a 770 × 770 pixel size. In Figure 4b, the *x* and *y* axes represent the image size, which in this case corresponds to a 1 mm^2^ image (1 pixel = 1.3 μm), while the *z* axis gives the frame number, acquisition time, and applied potential after considering the voltammetric scan rate.

**Figure 4:**
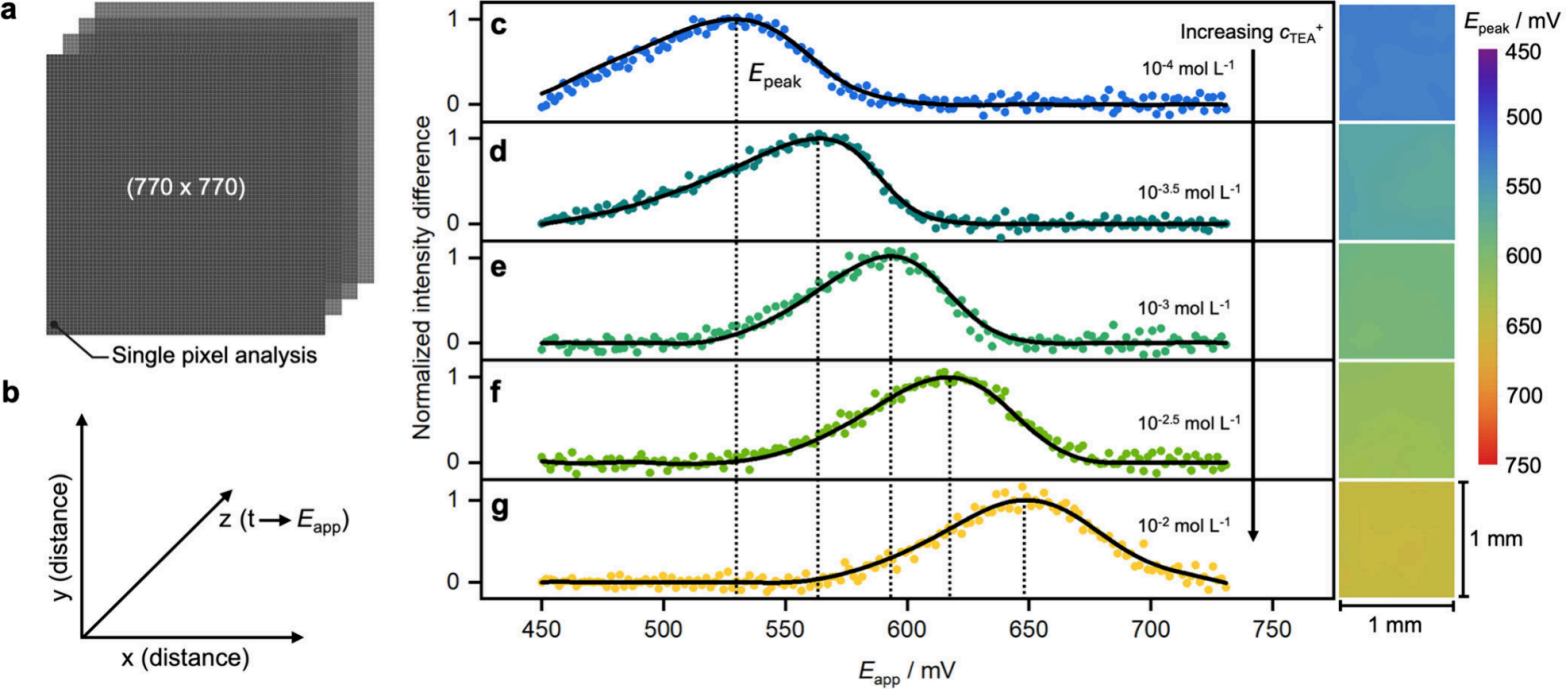
Single-pixel analysis for chemical mapping. (a and b) Scheme of the image stack for individual pixel analysis, where the *x* and *y* axes correspond to the image surface dimensions, while the *z* axis gives the position of each frame in time (*t*), which is then converted to applied potential (*E*_*app*_). (c–g) Normalized intensity difference for individual pixels from the image stacks generated during a potential sweep for different tetraethylammonium concentrations, which are shown in each panel. The data presented correspond to the pixel in the middle of each image stack, and the corresponding density plots (1 mm^2^) based on the transition potential (*E*_*peak*_) for each pixel are displayed in the right panel together with the false-color scale.

Figures 4c–g shows the normalized intensity differences for a *single* pixel in image stacks at different TEA^+^ concentrations, acquired during the voltammetric scans shown in Figure 3a. To compare, Supplementary Video 1 gives the raw experimental false-color image sequence for 10 mmol L^–1^ TEA^+^ for this experiment. During the image processing, a Gaussian filter function (solid lines) was applied to reduce noise in the discrete derivatives, and a peak-finding function determined the peak potentials (*E*_*peak*_). Based on the peak potential at which the optical transition is observed, a color was attributed to each pixel in the stack. A false-color density plot was then produced, corresponding to a logarithmic ion concentration map; see right panels of Figures 4c–g for different concentrations of TEA^+^ in solution (see Figure S3 for more detail). Since the ion is here homogeneously distributed in the solution in contact with the imaging film, the optical transition occurs at the same potential everywhere and the images are homogeneous (standard deviations of 1–2 mV).

### Revealing Invisible Ion Concentration Gradients in a Microfluidic Flowing Junction

The proposed imaging technique was further characterized under laminar flow conditions in which a junction of two contacting laminar solution flows of different compositions was evaluated by conventional wide-field fluorescence microscopy. Figure S4a shows a schematic of the electrochemical imaging cell built in-house. Two inlets feed different solutions into the channels that flow over the imaging electrode. In this study, solutions containing 1 mmol L^–1^ and 10 mmol L^–1^ of TEA^+^ in a 25 mmol L^–1^ of MgCl_2_ background electrolyte were introduced into the cell at a continuous linear velocity of 500 μm s^–1^, ensuring parallel solution flow. Figure S4b gives the detailed cell components and the inner volume domain of the microfluidic channels. The junction where the two channels merge aligns with the edge of the imaging film, which allows for direct, real-time visualization of the diffusional mixing between the two solutions.

Figure 5a presents the voltammetric scan recorded under controlled flow conditions within a potential range of 450 to 750 mV. Two distinct ion-transfer waves are visible, which suggests the electrochemical conversion of the TEMPO redox probe at two different potentials. This is consistent with the ion-selective sensing film being simultaneously exposed to two different TEA^+^ solution concentrations, resulting in two distinct phase-boundary potentials at the solution–membrane interface. The voltammogram gives aggregate information on the ion transfer properties of the film and does not offer spatially resolved information about ion distribution across the film.

**Figure 5:**
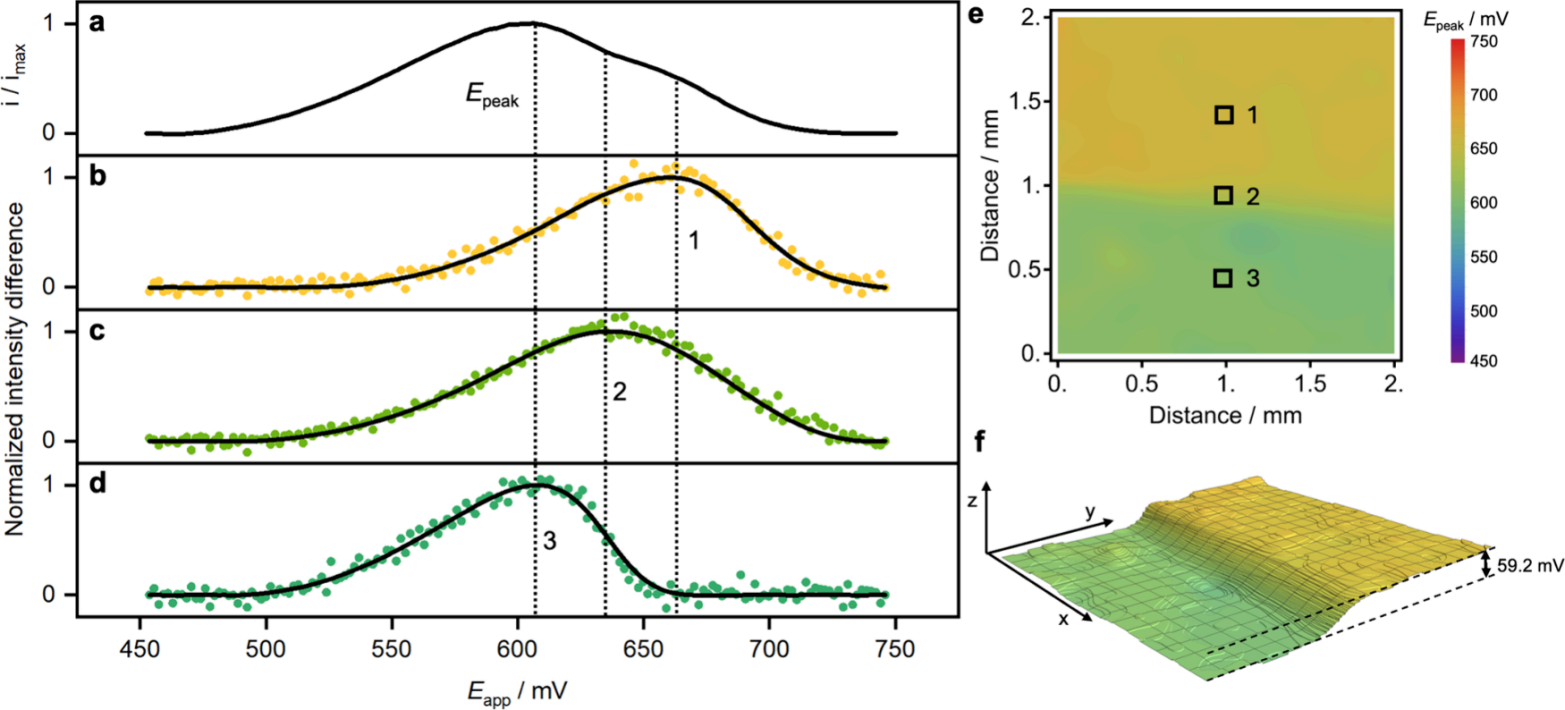
Electrochemical imaging of tetraethylammonium in a flow system. (a) Linear sweep voltammogram (15 mV s^–1^) for two TEA^+^ solutions (1 and 10 mmol L^–1^) flowing on the surface of the ion-selective membrane. (b–d) Normalized intensity difference data (symbols) and fitting curve (solid lines) for pixels from different regions of the image. (e) Density plot generated by the transition potential of each pixel in the image stack, with the corresponding false-color scale. (f) A 3D plot of the transition potential (same color scale) for all the pixels in the image with a potential step of 59.2 mV between the two flowing solutions. The *x* and *y* axes correspond to the image dimensions, and *z* is the axis for the applied potential.

In contrast, the novel imaging technique introduced here offers a direct means of visualizing the spatial distribution of the model tetraethylammonium ion by independently analyzing each pixel within the image stack generated during the potential scan. Figures 5b–d present the intensity difference curves obtained for three discrete pixel locations shown in Figure 5e, labeled as regions 1, 2, and 3. In this map, the electrochemical potential corresponding to the maximum *I*_*diff*_ (described in eq 2) can be visualized for all pixels within the applied potential window. This peak potential map is composed of more than 2 million pixels (1500 × 1500), for which data acquisition was completed in just 20 s, which corresponds to the duration of the voltammetric scan (300 mV window at 15 mV s^–1^). Given that each individual pixel represents an area of 1.3 μm × 1.3 μm, using a 5× objective, the entire image covers approximately 4 mm^2^, which is a significantly larger area compared to chemical maps obtained via conventional probing techniques.^6,46^

The observed shifts in the peak positions of the *I*_*diff*_ curves reflect the local ion concentration in each of the three designated regions and can be directly compared to theoretical expectations. Positions 1 and 3 correspond to the 10 mmol L^–1^ and 1 mmol L^–1^ TEA^+^ solutions, respectively, while position 2 is in the mixing zone between the two solution streams. The peak-finding function accurately determines the transition potentials for each pixel, yielding values of 662.8 mV for position 1 and 603.6 mV for position 3, resulting in a potential difference of 59.2 mV. This observed potential difference corresponds to the theoretical Nernstian potential step expected for a 10-fold concentration change.^37^ The pixel located at position 2 has a peak potential of 637 mV, consistent with the expected mixing region between the two solutions.

Figure 5f presents a 3D visualization of the transition map shown in Figure 5e where the peak potential transition region for the two flowing solutions is clearly distinguishable. One half of the image corresponds to the transition potential associated with the higher TEA^+^ concentration, while the other half shows the transition potential for the lower concentration. The color scale applied to the 3D plot follows the same scheme as in the 2D map in Figure 5e, allowing for a facile interpretation of the potential distribution. As expected, a potential step of 59.2 mV is observed between the two regions, confirming the theoretical predictions discussed previously.

Supplementary Video 2 shows the false-colored raw image stack used to generate the potential transition map shown in Figure 5e, corresponding to the flow experiment. The video shows how the signal intensity within the imaging region changes at different times in the applied potential window. It is possible to visualize the signal intensity increasing from the bottom part corresponding to the lowest TEA^+^ concentration, passing through the mixing region of intermediate ion concentration, and finally, the top part transitions at higher potentials corresponding to the highest TEA^+^ concentration.

Once the pump is stopped, steady-state laminar flow is stopped and the solutions start to mix. With time, a homogenization of TEA^+^ concentration over the membrane surface takes place. Figure S5a shows the voltammogram 3 min after stopping the flow. A single ion-transfer wave indicates a more uniform phase-boundary potential. Figure S5b and S5c show the corresponding 3D and 2D plots for flow-off after 3 min, visually illustrating the flattening of the peak potential owing to homogenization.

### Visualizing Localized Ion Concentration at High Spatial Resolution

The data presented in Figure 5e were converted into concentration information using the Nernstian slope from the peak potential of the entire image, giving the TEA^+^ concentration map shown in Figure S6 along with their mixing dynamics after the junction point in the flow cell, captured at 5× magnification. By further increasing the magnification to 10×, the spatial resolution was further enhanced to a submicrometer level, where each pixel in the image corresponds to 0.65 μm. The corresponding chemical image is shown in Figure 6a for a more detailed visualization of the concentration distribution within the mixing region. The corresponding peak potential map is shown in Figure S7. In addition to the resolution set by the microscopy itself, one may consider potential limitations arising from the diffusion behavior of the sensing and electrochemical components within the film. However, the film is only a few hundred nanometers thick, and the relevant diffusion coefficients are known to be 2 to 3 orders of magnitude lower than in the aqueous phase.^47^ This suggests that the ultimate diffusion limit of the film is expected to have a negligible influence when concentration gradients are imaged in solution.

**Figure 6:**
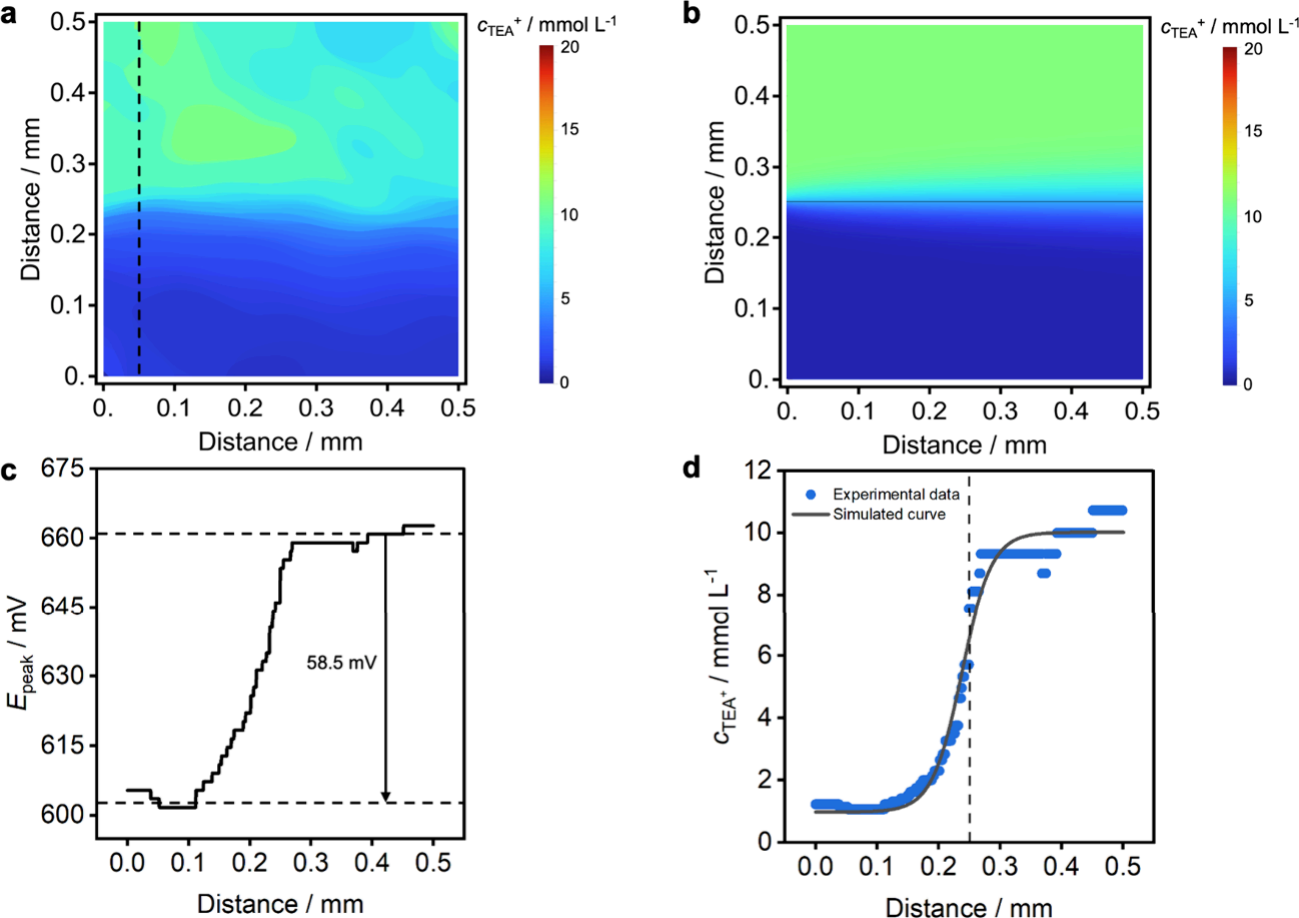
Electrochemically generated concentration map of tetraethylammonium. (a) Concentration map of two confluent TEA^+^ solution streams (1 and 10 mmol L^–1^) flowing over the surface of the imaging region, generated from an image stack acquired with 10× magnification. (b) Simulated concentration map showing TEA^+^ distribution in laminar flow with 10× magnification. (c) Transition potential profile sampled at 65 μm (along the *x*-axis) from the junction point, with an average potential step of 58.5 mV. (d) Corresponding experimental concentration profile (blue symbols) and simulated curve (solid black line) at 65 μm from the junction point showing the concentration gradients using 10× magnification.

To compare the experimentally acquired concentration maps with theoretical predictions, a simulation was performed by using the known geometry and linear flow velocity. The simulated concentration map, presented in Figure 6b, shows a good correlation with the experimental image in Figure 6a. A two-dimensional schematic representation of the microfluidic channels, along with the specific parameters used in the simulation, is provided in Figure S8.

To ensure that the proposed imaging technique can detect electrochemical changes at the single-pixel level, Figure 6c illustrates a two-dimensional transition potential profile across the imaging region (*y*-axis) at 65 μm (*x*-axis) from the junction point where the solutions meet, corresponding to Figure S7 at 10× magnification. As shown in the profile, the system is capable of resolving potential steps on the single-pixel scale. Furthermore, the total potential step within the distance range corresponds to 58.5 mV, which further confirms the Nernstian response slope of the approach. The same results converted to a concentration profile are shown in Figure 6d where the blue symbols are experimental data. At the steepest point of the curve, the concentration change corresponds to 1.4 mmol L^–1^ between two adjacent pixels, which ensures that this imaging system can recognize large concentration changes in short spatial steps.

The theoretical TEA^+^ concentration profile at a specific spatial position (along the *y*-axis) and time (*t*), resulting from the interdiffusion of the two confluent solution streams along the *x*-axis, was calculated by solving Fick’s second law for this situation:

3 $$c_{j^{+}}\left(y,t\right)=\left(\frac{c_{1}+c_{2}}{2}\right)+\left(\frac{c_{1}-c_{2}}{2}\right)\mathrm{erf}\left(\frac{y}{2\sqrt{Dt}}\right)$$

where *c*_1_ and *c*_2_ are the ion concentrations for both solutions, *D* is the diffusion coefficient of the species, and erf is the error function, with *t* transformed to distance under the known flow conditions. The calculated curve is shown as a black line in Figure 6d and again correlates well with the experimental data.

The nonidealities observed in the experimental chemical maps can be attributed to errors in determining the transition potential in the flow system. Although the ITO film deposited on glass is not perfectly homogeneous,^48,49^ potentially giving variations in potential over the electrode surface, this is unlikely to be the primary cause of deviations in the integrated flow system. This conclusion is supported by the chemical maps obtained under static conditions, which demonstrate higher homogeneity with standard deviations as low as 1.2 mV; see Figure 4.

To assess the uncertainty, a control experiment was conducted using two solutions of equal TEA^+^ concentration (1 mmol L^–1^ in the same background) in the flow cell. Figure S9 presents the corresponding voltammogram and 2D and 3D plots for peak potential and ion concentration for this homogeneous solution experiment. Some fluctuations are still evident, particularly in the density plots for both potential and concentration (Figures S9c and d). The potential and concentration values across the entire image were averaged perpendicular to the flow, giving the standard deviation for each pixel row in Figure S10. The primary source of error appears to be the potential drop in the flow cell, which increases with the electrode distance. From the Nernst equation, an uncertainty of just 1 mV already gives a concentration error of 4%, suggesting that the utmost care should be invested in improving the accuracy and reproducibility of the peak potential readings for this chemical imaging application.

## Conclusions

The imaging principle proposed here demonstrates significant promise in advancing the field of chemical imaging. For the first time, the electrochemical modulation of the redox state of a molecular probe has been shown to control the optical signal of an optical reporter. This innovation provides direct and spatially resolved insights into ion concentration distribution. The microfluidic device used to demonstrate the working mechanism allowed for the observation of ion diffusion dynamics at the single-pixel level, achieving sub-micrometer spatial resolution. Most importantly, the imaging platform based on TEMPO and rhodamine is compatible with commercial fluorescence microscopes without requiring additional modifications of the experimental setup, which opens the doors for numerous applications in biology and medicine. Challenges remain in addressing the error associated with identifying the peak potential owing to limited signal changes during the electrochemical modulation of fluorescence quenching. Future developments in the chemical toolbox could overcome this limitation. Moreover, prior research on multianalyte sensing with TEMPO^29^ suggests that imaging multiple ions within a single voltammetric scan on the same platform may soon be achievable, further expanding the potential of this technology.

## Supplementary Material







## Notes

Experimental section, mathematical script for image analysis, and additional ion concentration maps (PDF)
Video S1 (AVI) False-colored raw image stack for a homogenous ion distribution, used to generate the chemical image in Figure 4g (right panel).
Video S2 (AVI) False-colored raw image stack for two solution streams with different ion concentrations, used to generate the transition potential map in Figure 5e.

## Notes

A prior preprint of this work has been posted.^50^
