# Supplementary material for: Spatially Resolved Ion Sensing by Voltammetric Ion Transfer Microscopy
Source: JACS Au. 2025 Oct 21;5(11):5538–46. doi: 10.1021/jacsau.5c01034 (PMC12648306; doi:10.1021/jacsau.5c01034)
Supplement: Supplementary file 1 [file au5c01034_si_001.pdf]

Supporting Information for:

Spatially Resolved Ion Sensing by Voltammetric Ion Transfer Microscopy

Gabriel J. Mattos, Justine A. Rothen, Thomas J. Cherubini, and Eric Bakker*

Department of Inorganic and Analytical Chemistry, University of Geneva, 1211 Geneva, Switzerland
**corresponding author:* eric.bakker@unige.ch

Reagents

All aqueous solutions were prepared with deionized water (>18 MΩ cm). Magnesium chloride ($MgCl_2$), Tetraethylammonium nitrate ($TEANO_3$), rhodamine B octadecyl ester perchlorate ($C_{46}H_{67}ClN_2O_7$), tetrahydrofuran (THF), sodium tetrakis[3,5-bis(trifluoromethyl)phenyl]-borate (NaTFPB), high molecular weight poly(vinyl chloride) (PVC), bis(2-ethylhexyl)sebacate (DOS) were purchased from Sigma-Aldrich. Lip-TEMPO was synthesized as previously described [1]. The compound may be sourced from selectoprobe.com. Square indium tin oxide (ITO) coated glass slides (25 mm × 25 mm × 1.1 mm) with a surface resistivity of 8-12 Ω/sq were also acquired from Sigma-Aldrich.

Preparation of the imaging platform

To remove any residual organic contamination, indium tin oxide (ITO) coated glass slides (25 mm x 25 mm) were first immersed (10 min) in a surfactant solution, followed by sonication in deionized water, ethanol, and acetone (10 min in each solvent). The slides were finally dried under nitrogen flow.

The ion-selective membrane cocktail consists of equimolar concentrations of TEMPO and ion-exchanger (NaTFPB, 50 mmol kg^{-1}) and 25 mmol kg^{-1} of octadecyl ester rhodamine in a polymeric matrix based on polyvinyl chloride (PVC) and the plasticizer dioctyl sebacate (DOS), at a ratio of 1:3 (m/m). A total mass of 50 mg containing all the membrane components is dissolved in 1 mL of tetrahydrofuran (THF). The resulting cocktail (25 µL) is then used to spin coat a thin membrane on the ITO substrate, at a spinning rate of 1000 rpm for 2 min to allow the solvent to evaporate. According to ellipsometry measurements, the thickness of the membrane formed under these conditions was found to be 230 nm, which agrees with previous reports on this procedure [2].

A commercial transparent tape (Scotch 3M, crystal clear, 50 µm thickness) with a circular opening (∅3 mm) made with a punch hole was placed on top of the spin-coated membrane to delimit the electrode area. The modified ITO electrode was then placed in the electrochemical cell, together with an Ag/AgCl coated wire as a reference electrode, and a platinum wire served as the counter electrode.

The electrochemical flow cell for electrochemical imaging was built in-house to be used in commercially available fluorescence microscopes without further modifications. The microfluidic system was designed using SOLIDWORKS® CAD. Supplementary Fig. 4a is a scheme of the fully assembled cell. Supplementary Fig. 4b shows the exploded view of the cell components, where the base is made of plexiglass, a transparent tape (Scotch 3M, crystal clear, 50 µm thickness) is used as a membrane mask on top of the ITO. A layer of Mylar RS PRO (50 µm thick) containing a 3M™ Adhesive Transfer Tape 465 (50 µm thick) is used to seal the bottom of the fluidic path, which is made of one layer of Mylar RS PRO between two layers of a 3M™ Adhesive Transfer Tape 465 (50 µm thick). These two components are laser cut separately and assembled onto the cell. A rectangular (25 mm × 75 mm × 0.13 mm) glass coverslip closes the top of the cell, which has a total volume of 175 µL, calculated using COMSOL Multiphysics by creating an inner domain.

Image analysis routine

The image analysis procedure is fully automated on Wolfram Mathematica, as shown below for the image stack corresponding to a 1 mmol L^{-1} TEA^+ solution. Once the image stack is uploaded (SetDirectory), the individual name of each frame is given (FileNames) and only the frame number sequence remains (StringDelete), starting from 1 (iinit) till 161 (inumb), the total number of frames in the stack. Electrochemical parameters consist of the

initial potential in the scanning voltammogram (Einit) and the potential steps between the frames (dE), which depends on the scan rate and frame rate (experimental settings):

```
SetDirectory["XXXXXX"];

FileNames[]⟦1⟧

TEA30000.tif

iinit = 1;

Counter = StringDelete[FileNames[]⟦iinit⟧, {"TEA30", ".tif"}]

000

inumb = ToExpression[StringDelete[FileNames[]⟦-1⟧, {"TEA30", ".tif"}] ]

161
```

Electrochemistry parameters:

```
Einit = 450;
dE = 1.85;
```

The image dimensions (in y and x axes) in this case correspond to a 1 mm^2 image (770 pixels × 770 pixels):

This commands reveals the size of each image, in pixels:

```
xmax = ImageDimensions[Import[FileNames[]⟦iinit⟧]]⟦1⟧
ymax = ImageDimensions[Import[FileNames[]⟦iinit⟧]]⟦2⟧

770

770
```

The following procedure is then applied to identify the maximum fluorescence intensity difference:

Calculating the intensity difference between the frames:

```
For[u = iinit, u < inumb,
 i[u] = Import[FileNames[]⟦u⟧];
 idiff[u] = i[u] - i[u - 1];
 u++]
```

A lowpass filter is used for denoising data:

```
Do[idiff[u] = ImageData[LowpassFilter[i[u] - i[u - 1], 0.02]],
  {u, iinit + 1, inumb - 1}];

peakTEA3 = Table[Null, {k, 1, xmax}, {l, 1, ymax}];

data = ListPlot[Table[idiff[u]⟦350, 350⟧, {u, iinit + 1, inumb - 1}]];
```

Gaussian filter for smoothing the single pixel data and FindPeaks command to identify the peak signal change:

```
peaklist = FindPeaks[
   GaussianFilter[Table[idiff[u][[350, 350]], {u, iinit + 1, inumb - 1}], gf]];

peak = Part[Pick[Transpose[peaklist][[1]], y = Transpose[peaklist][[2]], Max[y]], 1];

fitting = ListLinePlot[
   GaussianFilter[Table[idiff[u][[350, 350]], {u, iinit + 1, inumb - 1}], gf],
   Prolog → {Line[{{peak, 0}, {peak, 0.003}}]},
   PlotStyle → Green];

Show[data, fitting, Prolog → {Line[{{peak, -0.1}, {peak, 0.003}}]},
 PlotStyle → Green, Frame → {{True, True}, {True, True}},
 BaseStyle → {20, FontFamily → "Arial"},
 AxesOrigin → {0, 0},
 ImageSize → {{500}, {500}},
 FrameLabel → {"Frame", "Intensity difference / a.u."},
 FrameStyle → Directive[Black, Thickness[0.008]], LabelStyle → Directive[Black]]
```

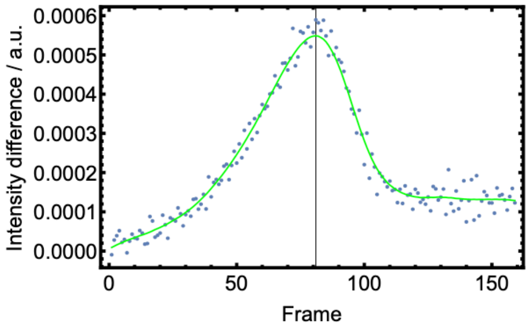

This procedure is then applied to every pixel in the image stack, giving the data used to generate the 3D and 2D plots of transition potential shown in this study.

Supplementary Video 1

False-colored raw image stack during the potential sweep from 450 mV to 750 mV (Figure 3a, 10 mmol L^{-1} TEA^{+}), used to generate the chemical image in Figure 4g (right panel).

Supplementary Video 2

False-colored raw image stack during the potential sweep from 450 mV to 750 mV in the microfluid flowing junction cell (Figure 5a, 1 and 10 mmol L^{-1} TEA^{+}), used to generate the transition potential map in Figure 5e and the concentration image in Supplementary Figure S6.

Describing the concentration of the quencher with the applied potential

The concentration of the quencher, TEMPO radical, in the imaging film, changes with the applied potential according to the theory for ion transfer voltammetry [3]. The time-dependent membrane potential of the imaging surface $E_m(t)$ consists of two contributions, the phase boundary potential corresponding to the ion (j^+) transfer across the membrane/aqueous sample solution interface, $\Delta_{aq}^{m}\phi_j(t)$, and the redox potential of the ion-to-electron transducer (TEMPO) between the ITO and the sensing film, $E_{TEMPO}(t)$:

$$E_m(t) = E_{TEMPO}(t) + \Delta_{aq}^{m}\phi_j(t) \quad (1)$$

In this ideal case, the electrochemical conversion of TEMPO is reversible and involves one electron, and the transferred ion is monovalent. Therefore, the Nernst equation for the time-dependent electron transfer potential and the concentrations of both oxidized and reduced forms of TEMPO in the imaging film is given as established:

$$E_{TEMPO}(t) = E^0_{TEMPO} + s\ log \frac{c^m_{TEMPO^+}(t)}{c^m_{TEMPO^\cdot}(t)} \tag{2}$$

Which can be simplified as:

$$\theta(t) = 10^{(E_{TEMPO}(t) - E^0_{TEMPO})/s} = \frac{c^m_{TEMPO^+}(t)}{c^m_{TEMPO^\cdot}(t)} \tag{3}$$

Where E^0_{TEMPO} is the standard potential for the TEMPO electron transfer process, s is the Nernstian slope, and the $\theta(t)$ function gives the ratio between the concentrations of the oxidized and reduced forms of TEMPO in the imaging film. Similarly, the ion transfer potential at the film/sample solution interface is given by:

$$\Delta^m_{aq}\phi_j(t) = \Delta^m_{aq}\phi^0_j + s\ log \frac{c^{aq}_j}{c^m_j(t)} \tag{4}$$

Which can also be simplified as:

$$\psi(t) = 10^{(\Delta^m_{aq}\phi_j(t) - \Delta^m_{aq}\phi^0_j)/s} = \frac{c^{aq}_j}{c^m_j(t)} \tag{5}$$

Where $\Delta^m_{aq}\phi^0_j$ is the standard potential for the ion transfer of the ion j across the imaging film/solution interface, c^{aq}_j is the concentration of the ion in the aqueous sample solution and $c^m_j(t)$ is the ion concentration in the imaging film. In this case, the concentration of the species in the imaging film is dictated by the electroneutrality of the film as the potential is applied during each voltammetric scan. As the concentration of the ion exchanger is the same as the TEMPO redox mediator, the concentration of the target ion in the film, $c^m_j(t)$, will be the same as the concentration of the quencher (TEMPO radical form) with time, $c^m_{TEMPO^\cdot}(t)$. By solving equations 3 and 5 for these variables and assuming the two terms are equal, it gives how the concentration of the oxidized form of the quencher changes with time, $c^m_{TEMPO^+}(t)$, which is the reciprocal of the corresponding reduced form $c^m_{TEMPO^\cdot}(t)$ within the applied potential window (E_{app}):

$$c^m_{TEMPO^\cdot}(t) = \frac{1}{c^{aq}_j} 10^{(E_{app} - E^0)/s} \tag{6}$$

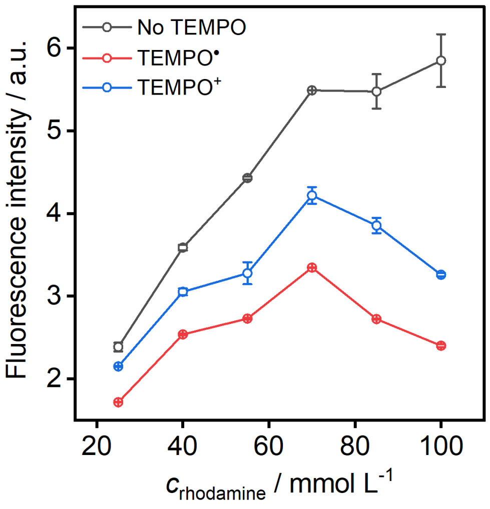

Figure S1. Optimization of the rhodamine concentration in the imaging film. The grey symbols represent the fluorescence intensity of the imaging film containing no TEMPO but the other sensing components: ion exchanger and changing concentrations of the optical reporter rhodamine in the polymeric matrix. Saturation of the signal is

observed in the range from 60 to 100 mmol L^{-1}. The red symbols show a significant decrease of the fluorescence intensity for different rhodamine concentrations in the presence of a constant concentration of the quencher, the TEMPO redox mediator in its radical form (25 mmol kg^{-1} $TEMPO^{\bullet}$ in the membrane cocktail). Blue symbols represent the fluorescence intensity after the quencher is electrochemically converted in its oxoammonium cation form ($TEMPO^{+}$), which also quenches the rhodamine fluorescence to some extent as the signal does not correspond to the membrane lacking TEMPO (grey symbols). This observation has been reported for other organic cations [4]. The concentration of 85 mmol L^{-1} rhodamine showed the greatest signal change with the electrochemical conversion of the TEMPO radical (40% signal increase), and, therefore, was used during all the experiments for ion imaging.

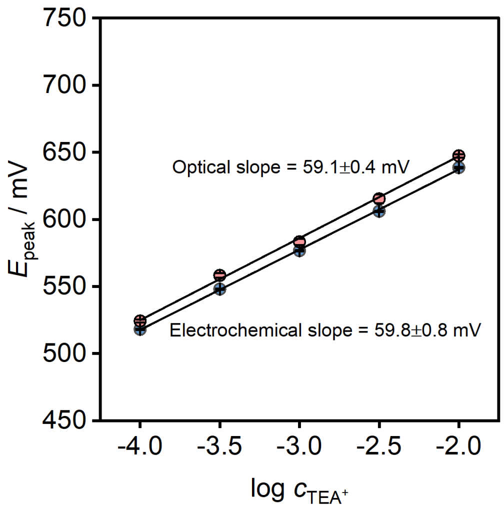

Figure S2. Opto-electrochemical calibration curves based on the peak potential of the ion transfer voltammograms and optical transition curves for TEA^{+} concentrations of 10^{-4}, $10^{-3.5}$, 10^{-3}, $10^{-2.5}$, and 10^{-2} mol L^{-1}. The corresponding slopes are shown next to the fitting lines.

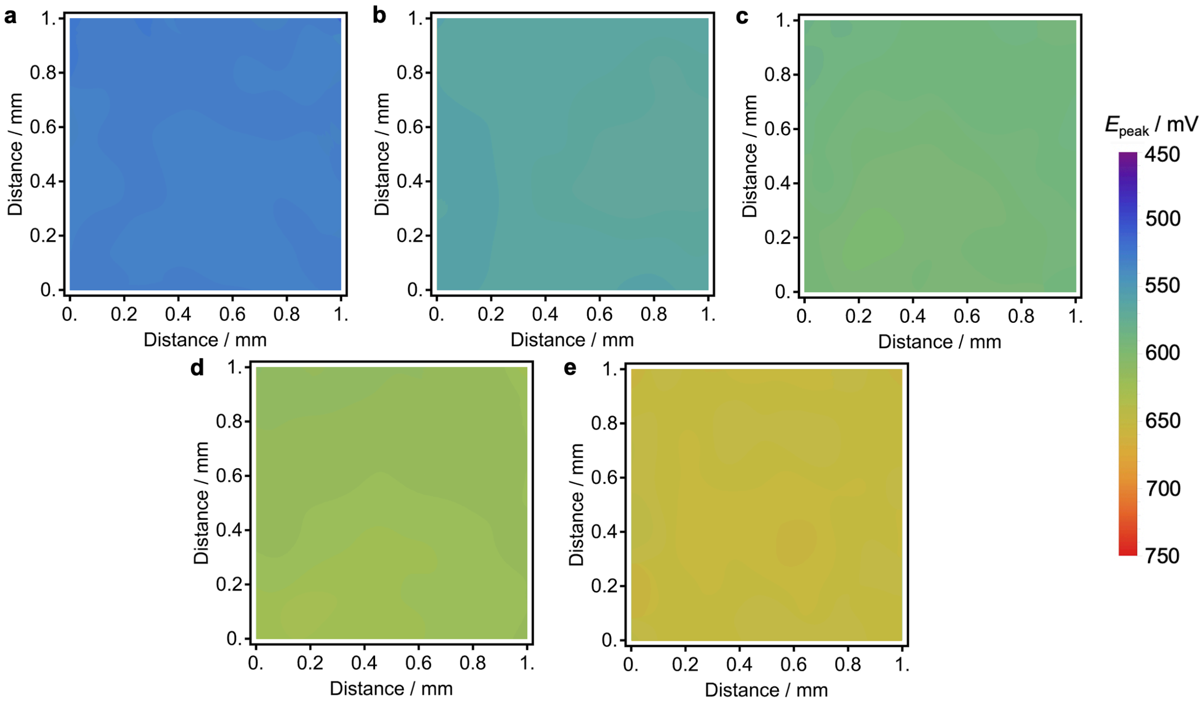

Figure S3. Density plots generated after determining the transition potential (E_{peak}) for every pixel in the image stacks acquired for different concentrations of tetraethylammonium in solution. (a-e), 10^{-4}, $10^{-3.5}$, 10^{-3}, $10^{-2.5}$, and 10^{-2} mol L^{-1}.

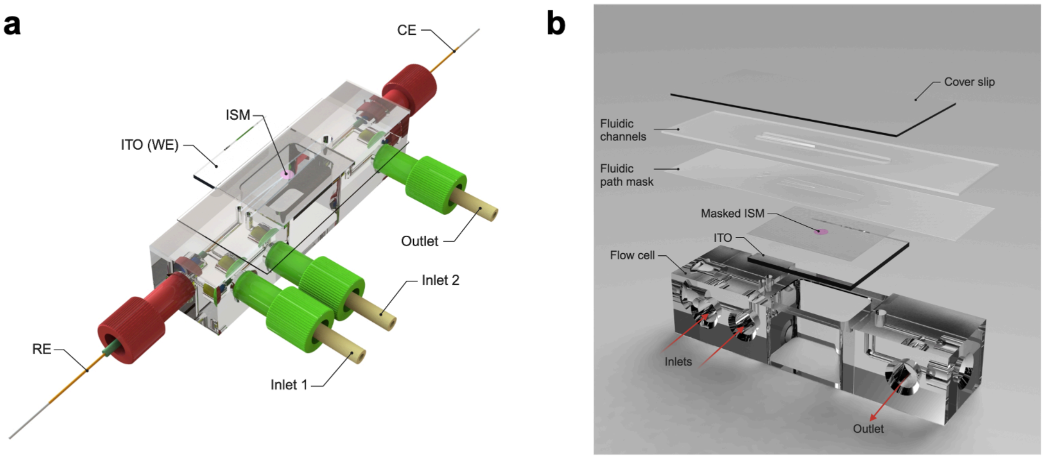

Figure S4. (a) Schematic view of the integrated microfluidic electrochemical cell used for ion microscopy experiments, containing: two inlets, one common outlet, working (WE), reference (RE), and counter (CE) electrodes. The ion-selective membrane (ISM) is the imaging/sensing surface. (b) Disassembled view of the flow cell components.

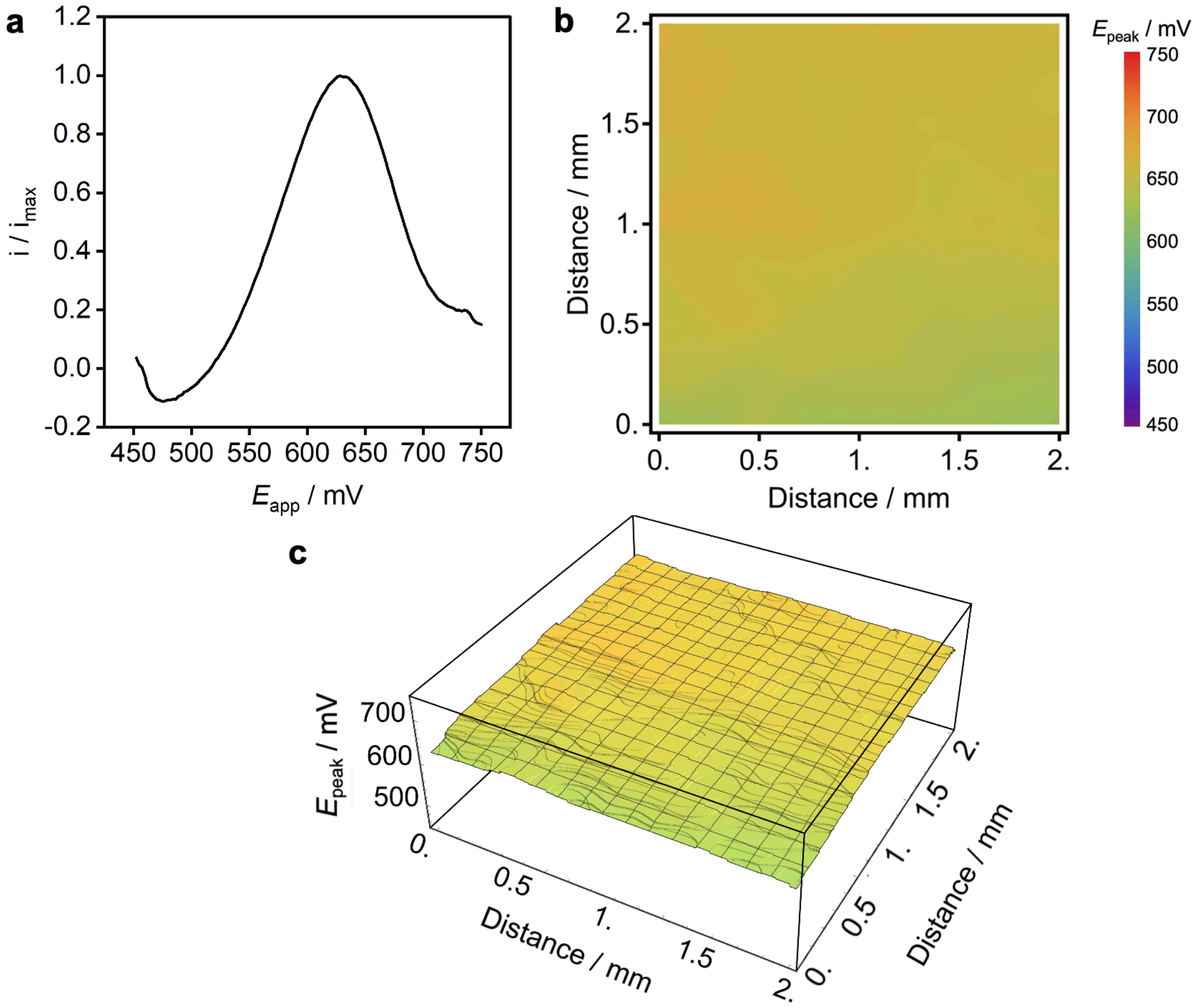

Figure S5. (a) Linear sweep voltammogram (15 mV s^{-1}) acquired 3 minutes after stopping the flow of the two TEA^+ solutions (1 and 10 mmol L^{-1} on the surface of the ion-selective membrane. (b and c**),** 2D and 3D transition potential maps generated 3 minutes after the laminar flow was stopped.

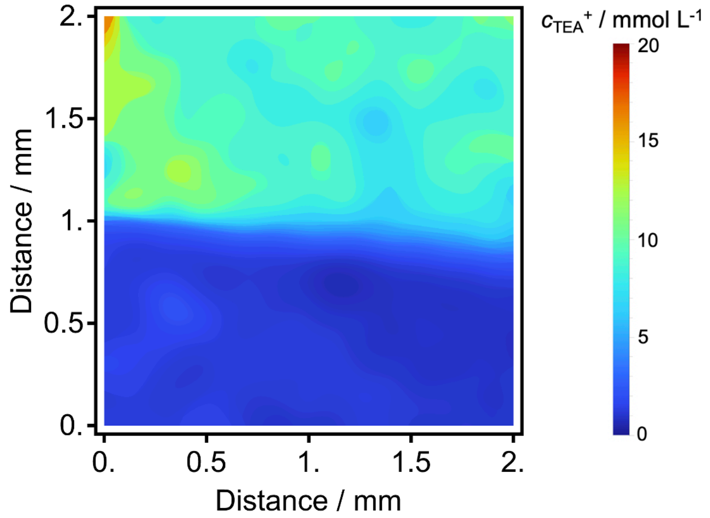

Figure S6. Concentration map of two confluent TEA^+ solution streams (1 and 10 mmol L^{-1}) flowing over the surface of the imaging region, generated from an image stack acquired with 5× magnification.

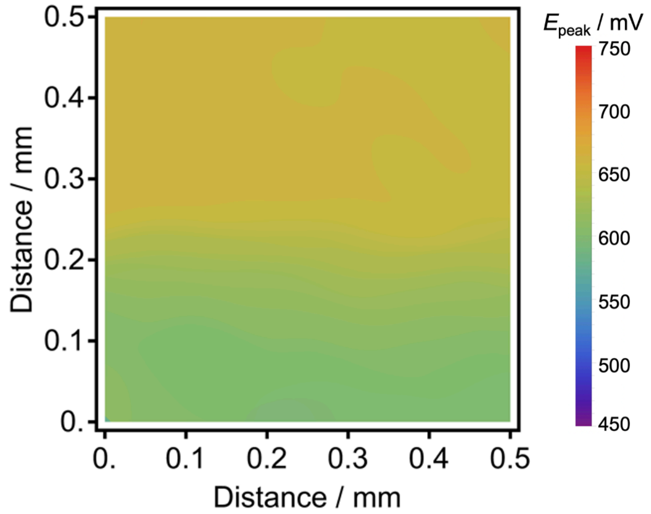

Figure S7. Transition potential map at 10× magnification for the two confluent TEA^+ solution streams (1 and 10 mmol L^{-1}).

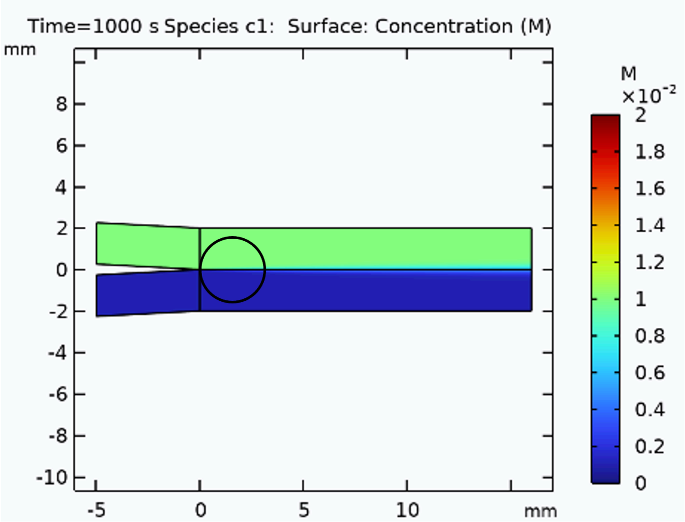

Figure S8. 2D scheme of the simulated flow (v = 500 µm s^{-1}) in the microfluidic channels for the two TEA^+ solutions (1 and 10 mmol L^{-1}, D = 1.2 × 10^{-9} m^2/s), performed on COMSOL Multiphysics. The geometry used represents the flowing junction in the cell, where the circle corresponds to the imaging area.

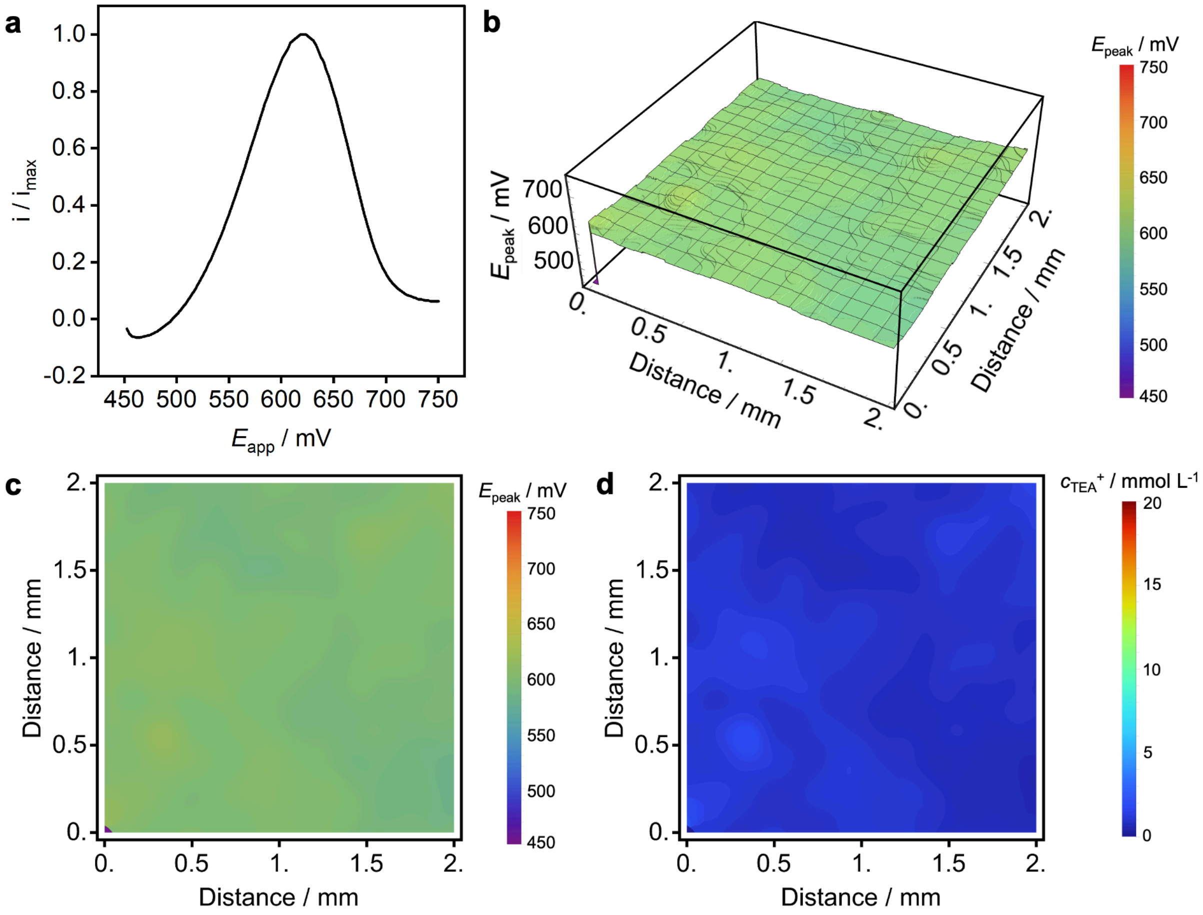

Figure S9. (a) Linear sweep voltammogram (15 mV s^{-1}) when 1 mmol L^{-1} TEA^+ solutions are flowing in both channels of the flow cell. (b) Corresponding 3D plot of the transition potential for all the pixels across the imaging surface. (c and d), 2D potential transition and concentration maps for the homogenous distribution of TEA^+ in the flowing junction system.

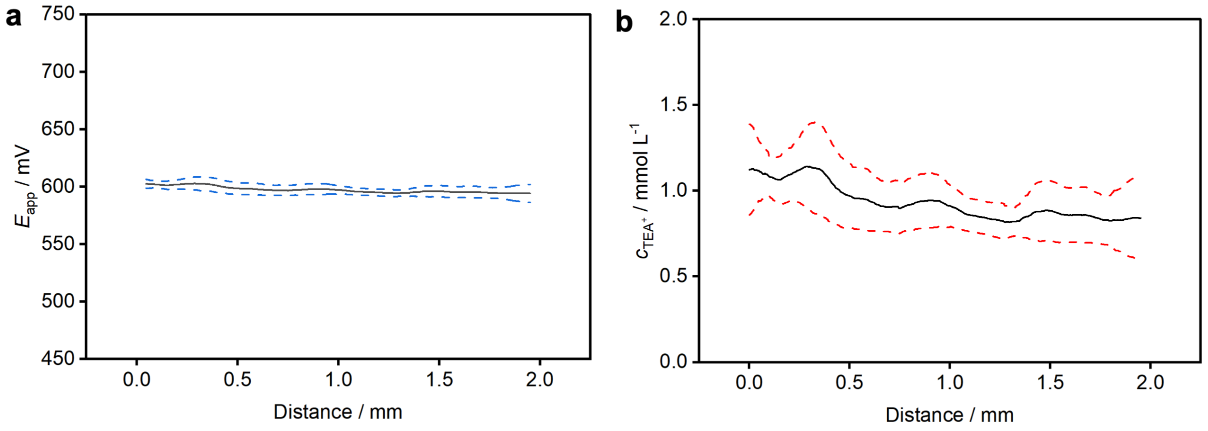

Figure S10. (a) Average transition potential (black solid line) along the 1500 pixel columns (2.0 mm) with the associated standard deviation (blue dashed lines) for a homogenous distribution (1 mmol L^{-1}) of the model ion on the imaging surface in the flow cell system. (b) Corresponding average concentration of tetraethylammonium (solid black line) with the associated standard deviation (red dashed lines). The concentration error considering all the pixels across the image is -6.8%.

References

(1) Mattos, G. J.; Tiuftiakov, N. Yu.; Bakker, E. Lipophilic Tetramethylpiperidine N-Oxyl (TEMPO) as a Phase-Transfer Redox Mediator in Thin Films for Anion and Cation Sensing. *Electrochemistry Communications* **2023**, *157*, 107603. https://doi.org/10.1016/j.elecom.2023.107603.

(2) Crespo, G. A.; Cuartero, M.; Bakker, E. Thin Layer Ionophore-Based Membrane for Multianalyte Ion Activity Detection. *Anal. Chem.* **2015**, *87* (15), 7729–7737. https://doi.org/10.1021/acs.analchem.5b01459.

(3) Mao, C.; Yuan, D.; Wang, L.; Bakker, E. Separating Boundary Potential Changes at Thin Solid Contact Ion Transfer Voltammetric Membrane Electrodes. *Journal of Electroanalytical Chemistry* **2021**, *880*, 114800. https://doi.org/10.1016/j.jelechem.2020.114800.

(4) Bertocchi, M. J.; Lupicki, A.; Bajpai, A.; Moorthy, J. N.; Weiss, R. G. Influence of Cations on the Fluorescence Quenching of an Ionic, Sterically Congested Pyrenyl Moiety by Iodide in Water. *J. Phys. Chem. A* **2017**, *121* (40), 7588–7596. https://doi.org/10.1021/acs.jpca.7b07853.
